# A Cytoplasmic Negative Regulator Isoform of ATF7 Impairs ATF7 and ATF2 Phosphorylation and Transcriptional Activity

**DOI:** 10.1371/journal.pone.0023351

**Published:** 2011-08-16

**Authors:** Jessica Diring, Barbara Camuzeaux, Mariel Donzeau, Marc Vigneron, Manuel Rosa-Calatrava, Claude Kedinger, Bruno Chatton

**Affiliations:** 1 Université de Strasbourg, UMR7242 Biotechnologie et Signalisation Cellulaire, Ecole Supérieure de Biotechnologie de Strasbourg, BP10413, Illkirch, France; 2 Laboratoire de Virologie et Pathologie Humaine VirPath, Université Claude Bernard Lyon 1, Hospices Civils de Lyon, Lyon, France; Université Paris-Diderot, France

## Abstract

Alternative splicing and post-translational modifications are processes that give rise to the complexity of the proteome. The nuclear ATF7 and ATF2 (activating transcription factor) are structurally homologous leucine zipper transcription factors encoded by distinct genes. Stress and growth factors activate ATF2 and ATF7 mainly via sequential phosphorylation of two conserved threonine residues in their activation domain. Distinct protein kinases, among which mitogen-activated protein kinases (MAPK), phosphorylate ATF2 and ATF7 first on Thr71/Thr53 and next on Thr69/Thr51 residues respectively, resulting in transcriptional activation. Here, we identify and characterize a cytoplasmic alternatively spliced isoform of ATF7. This variant, named ATF7-4, inhibits both ATF2 and ATF7 transcriptional activities by impairing the first phosphorylation event on Thr71/Thr53 residues. ATF7-4 indeed sequesters the Thr53-phosphorylating kinase in the cytoplasm. Upon stimulus-induced phosphorylation, ATF7-4 is poly-ubiquitinated and degraded, enabling the release of the kinase and ATF7/ATF2 activation. Our data therefore conclusively establish that ATF7-4 is an important cytoplasmic negative regulator of ATF7 and ATF2 transcription factors.

## Introduction

The characterization of cellular pathways leading to all the post-translational modifications of a protein is essential to understand the molecular mechanisms regulating its functions. Crosstalks between different types of such modifications are an emerging theme in eukaryotic biology. Thus, examples of multiple connections between phosphorylation, sumoylation and ubiquitination have been described (for a review see [1]). Within the AP-1 transcription factors family, the ATF2, ATF7 and CREB5 compose a subfamily based on sequence conservation [2], [3], [4], [5]. The transcriptional activation and DNA-binding domains of ATF2 and ATF7 factors are highly conserved and their specificity is mainly governed by post-translational modifications and interactions with specific cofactors [6], [7], [8], [9]. ATF2 is a protein that displays diverse, tissue-dependent functions [10], [11]. For instance, ATF2 has been implicated in malignant and non-malignant skin tumor development [12], [13]. ATF2 also elicits a suppressor function in mammary tumors [14], and mediates lipopolysaccharide-induced transcription in macrophage cells [15].

ATF7 shares a considerable sequence homology with ATF2, especially within the C-terminal DNA-binding/dimerization domain and the N-terminal activation domain. This latter region includes a critical zinc-binding element and two conserved threonine residues (Thr51 and Thr53 corresponding to the Thr69 and Thr71 homologues in ATF2). Different mitogen-activated protein kinases (MAPK), particularly members of JNK and p38 families, specifically phosphorylate these conserved threonine residues of ATF2 and ATF7 leading to transcriptional activation [16], [17], [18], [19], [20], [21], [22], [23], [24], [25]. These phosphorylation events are essential for ATF7/ATF2 proteins function *in vivo*, since Atf2^A/A^ mice, carrying mutations in these critical phosphorylation sites, have a strong phenotype, identical to that seen upon deletion of the DNA-binding domain [26], [27]. Combining this mutant with a knockout of ATF7 (Atf7^−/−^) results in embryonic lethality with severe abnormalities in the developing liver and heart [26]. Moreover, Atf7^−/−^ mice exhibit abnormal behaviors and increased serotonin 5-HT receptor 5B (Htr5b) mRNA levels in the dorsal raphe nuclei [28].

Alternative splicing of pre-mRNAs accounts for a large proportion of proteomic complexity [29], [30]. New splicing events occurring within protein coding regions potentially alter the biological function of the gene by expressing polypeptides with novel functions [31], [32]. Until now, the ATF7 transcription factor subfamily was known to include at least two major proteins (ATF7-1, ATF7-2) translated from alternatively spliced messengers transcribed from a unique gene [33]. In the present study, we report the identification and functional characterization of a short alternatively spliced variant of ATF7 that is highly conserved amongst mammalian species, except mouse and rat. This messenger RNA encodes a functional C-terminally truncated protein, ATF7-4, whose intracellular location is restricted to the cytoplasm and which downregulates the transcriptional activity of both nuclear ATF7 and ATF2 transcription factors. We show that, under resting conditions, ATF7-4 interacts in the cytoplasm with a kinase. Our data are compatible with the idea that this kinase is responsible for the priming phosphorylation event of the ATF7/ATF2 factors. In contrast, a stimulus-induced phosphorylation of ATF7-4 leads to its poly-ubiquitination and degradation by a proteasome 26S-dependent pathway, enabling ATF7/ATF2 activation.

## Results

### Identification of a new ATF7 isoform

**Figure 1:**
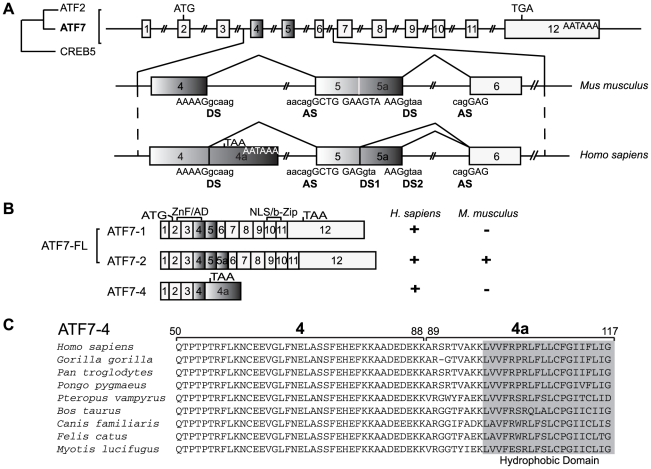
Identification of ATF7-4 as a novel alternatively spliced ATF7 isoform. (A) The structure of the ATF7 gene, a member of the ATF2/ATF7/CREB5 family, is shown schematically: the twelve exons are indicated by numbered boxes, and an alternative splicing region encompassing exons 4 to 6 is compared between human and mouse species. The sequences of the donor (DS) and acceptor (AS) sites for splicing are indicated, capital and small letters corresponding to the exonic and the intronic parts respectively. 4a and 5a are alternatively included exons adjacent to exons 4 and 5 respectively. The start codon, stop codons and polyadenylation sites are shown. (B) Schematic representation of the exonic structure of three ATF7 alternatively spliced isoforms. The regions encoding the zinc finger (ZnF)/activation domain (AD) or the nuclear localization signal (NLS)/basic region-leucine zipper (b-Zip) are indicated. “**+**” indicates that the proteins are encoded, either in human or in mouse species. (C) Amino acid sequence alignment of ATF7-4 exon 4 and specific exon 4a of a series of mammalian species. A conserved C-terminal hydrophobic domain is highlighted.

To look for new ATF7 isoforms that could participate in the ATF7-dependent transcriptional regulation, we performed anchored RT-PCR on HeLa mRNA using oligo-(dT) and ATF7-specific primers overlapping the translation initiation codon. We obtained a short amplification product (809 bp) that encodes an ATF7 alternatively spliced isoform of 117 residues designated ATF7-4 (following the ATF7 nomenclature). However, no such short product was amplified in mouse 3T3 fibroblasts under the same conditions (data not shown). To understand the existence of this new isoform, a comparative analysis of human and mouse ATF7 gene sequences was performed (Figure 1A). First, we observed that the human exon 4 is followed by a potential exon, referred to as exon 4a, that contains an open-reading frame encoding 29 amino acids. Moreover, this newly identified exon that is not found in the mouse gene displays a polyadenylation sequence (AATAAA) at its 3′ border. The inclusion of exon 4a is enabled when the splice donor site (DS) of exon 4 is not used. As a result, a short transcript encoding ATF7-4 is generated. Interestingly, examination of the nucleotide sequence of the ATF7 gene also revealed in exon 5 an alternative splice donor site (DS1), absent in the mouse gene, that can be used in place of the donor site of the exon 5a (DS2) (Figure 1A). The use of DS1 gives rise to the human ATF7-1 isoform. By contrast, the ATF7-2 variant encompassing exons 5 and 5a is expressed in both human and mouse. Thus, three major different transcripts are encoded in human (ATF7-1, ATF7-2, ATF7-4) whereas only one (ATF7-2) is encoded in mouse (Figure 1B).

The ATF7-4 protein contains the common ATF7 N-terminal moiety (88 residues) encompassing the transcriptional activation domain [34], and a 29-amino acid extension fused in frame. However, it lacks the entire C-terminal basic region and leucine zipper domain (residues 89–494) that function in ATF7-1 and ATF7-2 as a DNA binding domain/nuclear localization signal (NLS) and as a dimerization domain respectively [33] (Figure 1B). It is likely therefore that ATF7-4 exhibits functions that are distinct from those of the ATF7 full-length (ATF7-FL) isoforms. The alignment of peptide sequences derived from genomic sequences available in the databases reveals that the ATF7-4 isoform is potentially encoded in many mammalian genomes but not in mouse or rat. Moreover, it exhibits a highly conserved hydrophobic domain (HD) in its C-terminal moiety (Figure 1C).

### ATF7-4 is ubiquitously but differentially transcribed

**Figure 2:**
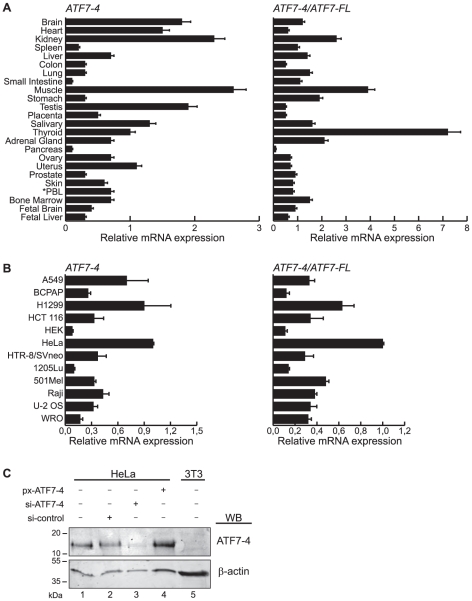
Pattern of expression of ATF7-4 transcript. (A and B) Real-time RT-PCR analysis of ATF7-4 gene expression profile (left panels) (A) in a 24 human tissue-cDNA array and (B) in a selection of human transformed cell lines. Right panels show the ATF7-4 expression relative to that of ATF7-FL. PBL stands for peripheral blood leucocytes. Data are the average of at least five independent experiments (standard deviations are shown). Data were normalized to β-actin gene expression. (C) Endogenous ATF7-4 protein was analyzed by western-blotting (WB) with a specific anti-ATF7-4 monoclonal antibody in human HeLa cells and mouse 3T3 fibroblasts. HeLa cells were transfected with either the px-ATF7-4 expression vector or a control, or specific siRNA targeting ATF7-4. β-actin was analyzed in parallel as a loading control.

To investigate the expression pattern of the ATF7-4 isoform, cDNAs panels from human tissues and different cell lines were analyzed by qRT-PCR using primers that are specific of the different species of ATF7 cDNAs (Table S1). We observed that ATF7-4 and ATF7-FL (i.e. ATF7-1 and ATF7-2) transcripts are detectable in all the human tissues and cell lines tested, reflecting a ubiquitous expression (Figure 2A and B, left panels, and Figure S2). However, the ATF7-4 transcript is predominantly detected in brain, heart, kidney, muscle, testis and also in HeLa, A549 and H1299 cell lines (Figure 2A and B, left panels). As expected, no ATF7-4 expression was detected in mouse embryonic fibroblasts (MEF, data not shown). We then assessed the relative ratio of ATF7-4 mRNA levels compared to those of ATF7-FL in the same tissues and cell lines. Interestingly, this analysis revealed a rather strong disparity of the relative accumulation levels of the two mRNA species, ATF7-4 mRNAs being two to seven times more abundant in some organs, i.e. kidney, muscle, thyroid, and nearly absent in pancreas (Figure 2A and B, right panels).

ATF7-4 protein level was next analyzed by western-blot in HeLa and 3T3 cell extracts (Figure 2C). The monoclonal antibody used in these experiments was directed against a peptide spanning residues 96–108, a region unique to the ATF7-4 protein. As expected, ATF7-4 protein expression was detected in HeLa but not in mouse 3T3 cell extracts (Figure 2C). Moreover, a specific siRNA efficiently knocked down ATF7-4. Together, these results indicate that human ATF7-4 is ubiquitously but differentially expressed, potentially suggesting a tissue-specific role for this variant. The high ATF7-4 mRNA and protein levels detected in HeLa cells led us to study its cellular function in those cells.

### ATF7-4 is localized in the cytoplasm

**Figure 3:**
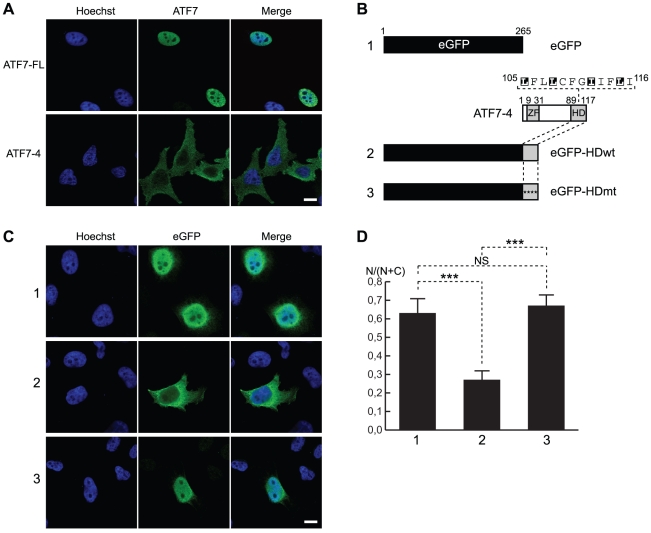
The hydrophobic domain of ATF7-4 controls its cytoplasmic localization. (A and C) Confocal microscopy images of (A) ATF7-FL, ATF7-4, and (C) eGFP proteins or eGFP-ATF7-4 fusion versions overexpressed in HeLa cells and stained by immunofluorescence with specific antibodies (central panels). DNA was stained with Hoechst 33258 (left panels). A merge of signals is shown on the right panels. (B–D) Analysis of the functional role of the ATF7-4 hydrophobic domain. (B and C) The relocalization of eGFP (lane 1) was assessed for eGFP fusion proteins with ATF7-4 hydrophobic domain (HD), either wild type (wt, lane 2) or mutant version (mt, lane 3). In the latter construct, the four conserved hydrophobic residues (highlighted) were mutated (asterisks) to alanine. Representative confocal images are shown. (D) The fluorescence intensity in the nucleus (N) over the total cell intensity (N+C) was quantified using ImageJ. The results presented are the average of at least 22 cells analyses for each condition. Standard deviations and statistical significance by Student t-test are shown: NS = nonsignificant, ***p<0.001. Scale bar: 10 µm.

Since ATF7-4 essentially comprises the activation domain of the ATF7 proteins and lacks their C-terminal dimerization/nuclear localization signal (NLS) domain, we examined its subcellular localization compared to those of the ATF7-FL proteins. Immunofluorescent staining experiments with specific ATF7 antibodies revealed that overexpressed ATF7-4 protein mainly localizes in the cytoplasm, whereas ATF7-FL proteins are nuclear in HeLa cells (Figure 3A). In an attempt to map the peptidic elements mediating ATF7-4 cytoplasmic localization, the hydrophobic domain (HD) that is specific to ATF7-4 and absent in ATF7-FL was fused, either in its wild-type or mutant version, at the C-terminal end of the eGFP (Figure 3B). In the mutant derivative, the hydrophobic domain was disrupted by replacing four leucine and isoleucine residues with alanine residues (Figure 3B). These eGFP-fusion proteins were then expressed in HeLa cells, and their localization was assessed by immunofluorescence (Figure 3C). A quantification of the nuclear fluorescence intensity over the total cell intensity was performed for each construct (Figure 3D). Control experiments indicated that the eGFP protein was expressed both in nucleus and cytoplasm (Figure 3C and D, row 1), whereas it was clearly directed to the cytoplasm when fused to the ATF7-4 specific domain (Figure 3C and D, row 2). Moreover, the nuclear localization was rescued by the mutations in the hydrophobic domain (Figure 3C and D, row 3). These results clearly demonstrate that the ATF7-4 transcription factor isoform is cytoplasmic, and that this localization is mediated by its C-terminal hydrophobic domain.

### ATF7-4 is phosphorylated and subsequently degraded by a proteasome-dependent pathway

**Figure 4:**
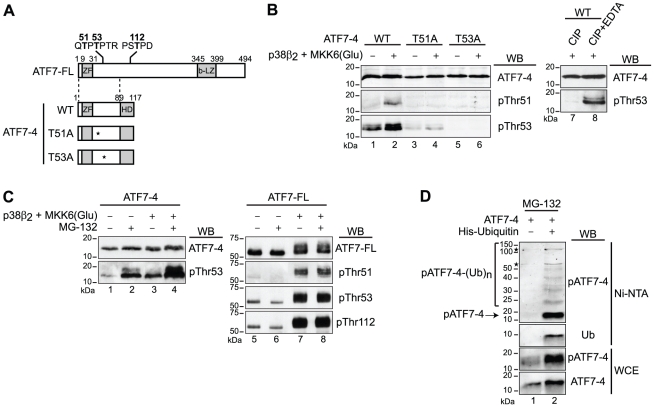
ATF7-4 is phosphorylated and subjected to degradation by a proteasome 26S-dependent pathway. (A) Schematic diagrams indicating the known phosphorylated residues of ATF7-FL protein and the derived series of ATF7-4 mutants used. (B) The indicated WT or mutant versions of ATF7-4 were co-expressed with p38β_2_ and constitutively active MKK6(Glu) in HeLa cells. Cell lysates were either untreated or treated with calf intestinal phosphatase (CIP), in presence of EDTA where indicated. The analysis was performed by western-blotting (WB) with specific antibodies as specified to detect non-phosphorylated (ATF7-4) and phosphorylated ATF7-4, either on residues Thr51 (pThr51) or Thr53 (pThr53). (C) ATF7-4 (lanes 1–4) and ATF7-FL (lanes 5–8) were co-expressed with p38β_2_ and MKK6(Glu) in HeLa cells treated with MG-132 proteasome inhibitor as indicated. Cell lysates were analyzed by WB with specific antibodies to detect ATF7-4, ATF7-FL and their phosphorylated forms. (D) ATF7-4 was co-expressed with 6His-Ubiquitin in HeLa cells treated overnight with MG-132. Whole cell extracts (WCE) were purified by a nickel affinity pulldown (Ni-NTA) and analyzed by WB with specific antibodies to detect the poly-ubiquitinated forms of ATF7-4. The asterisks indicate nonspecific signal.

We have previously demonstrated that both basal and p38β_2_ MAP kinase-stimulated ATF7-FL transcriptional activities depend mainly on the phosphorylation of residues Thr51, Thr53 and Thr112 within the activation domain of the protein, although the p38β_2_-mediated phosphorylation is restricted to Thr51 [23]. Since ATF7-FL proteins and the cytoplasmic variant ATF7-4 share the same N-terminal part of the activation domain, we investigated the contribution of the two Thr51 and Thr53 residues in both basal and p38β_2_-mediated ATF7-4 phosphorylation. Site-directed mutagenesis was performed to modify these two residues into alanine residues, generating the T51A and T53A mutants (Figure 4A). The wild-type or mutated versions of ATF7-4 were co-expressed or not with MKK6-activated p38β_2_ in HeLa cells and analyzed by western-blotting using specific antibodies. The monoclonal antibody directed against the C-terminal part of ATF7-4 specifically recognizes non-phosphorylated ATF7-4 (Figure 4B, upper panel, and data not shown). The phospho-specific antibodies targeting ATF7 when it is phosphorylated on Thr51 (pThr51) and Thr53 (pThr53) have been previously described [23] (Figure 4B, lower panels). In the absence of activated p38β_2_, the phosphorylation of Thr53 residue could be revealed using the pThr53 antibody (Figure 4B, lane 1). However, no signal was detected with the pThr51 antibody, indicating that, like the ATF7-FL protein, ATF7-4 is not phosphorylated on residue Thr51 under these conditions. By contrast, co-expression of activated p38β_2_ led to Thr51 phosphorylation (Figure 4B, lane 2). This double phosphorylated form (Thr51/Thr53) was more efficiently recognized by the pThr53 antibody, as previously described [23], resulting in an increased signal (Figure 4B, lane 2). The mutation of each of the two threonine residues affected the phosphorylation pattern of the ATF7-4 protein (Figure 4B, lanes 3–6). Importantly, the T53A mutation impaired Thr51 phosphorylation (Figure 4B, lane 6), indicating that Thr53 integrity is required for subsequent Thr51 phosphorylation, as it is the case for ATF7-FL that exhibits a two-step phosphorylation mechanism [23]. That the ATF7-4 species detected by the phospho-specific antibodies correspond to phosphorylated forms is further demonstrated by their sensitivity to calf intestinal phosphatase (CIP) treatment and their rescue in presence of EDTA, an inhibitor of CIP (Figure 4B, lanes 7 and 8). In conclusion, the ATF7-4 protein is phosphorylated in a two-step manner like ATF7-FL, first on Thr53 and then on Thr51 by the p38β_2_ MAP kinase.

We next studied the stability of ATF7-4 and ATF7-FL proteins by using MG-132, a proteasome 26S inhibitor. ATF7-4 (Figure 4C, lanes 1–4) or ATF7-FL (Figure 4C, lanes 5–8) proteins were co-expressed or not with activated p38β_2_ in HeLa cells in the absence or presence of MG-132. We then compared the respective amounts of the non-phosphorylated and phosphorylated ATF7-4 and ATF7-FL proteins in these conditions. After MG-132 treatment, we observed a nearly ten fold enrichment of both basal phosphorylated (Figure 4C, lane 2) and p38β_2_-phosphorylated (Figure 4C, lane 4) ATF7-4 proteins in cell extracts, clearly indicating that phosphorylated ATF7-4 is stabilized after proteasome inhibition. In contrast, no difference could be observed for non-phosphorylated ATF7-4 (Figure 4C, lanes 1–4, upper panel) or ATF7-FL (Figure 4C, lanes 5–8), whether phosphorylated or not, under the same conditions. Thus, it appears that the ATF7-4 isoform is specifically prone to proteasome-dependent degradation once phosphorylated, in contrast to ATF7-FL.

**Figure 5:**
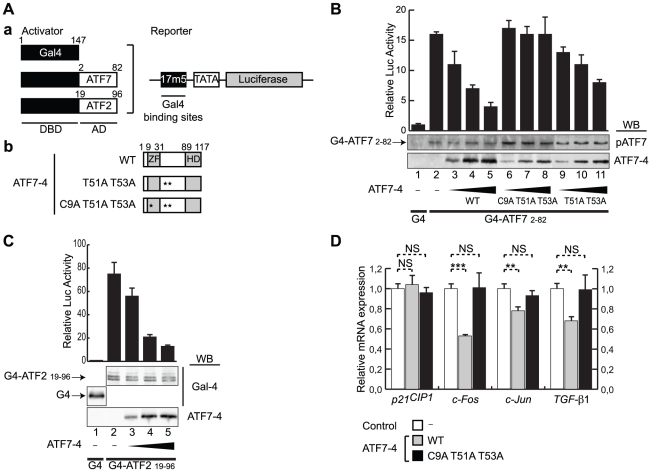
ATF7-4 inhibits the transcriptional activity of ATF7-FL and ATF2. (A–C) (A) (a) The luciferase activity assays were performed by co-expressing a Gal4-dependent luciferase reporter and Gal4-ATF fusion versions in HeLa cells. Gal4 DNA binding domain (DBD) was fused to the activation domains (AD) of either ATF7 (B) or ATF2 (C). (A) (b) The transcriptional activities were measured in presence of increasing amounts of co-expressed ATF7-4 or mutant versions. (B and C) Cell lysates were assayed for luciferase activity and analyzed in parallel by western-blotting (WB) with specific antibodies. The data of representative experiments are presented (standard deviations are shown). (D) Endogenous ATF7-FL/ATF2 transcriptional activity was measured by a real-time RT-PCR analysis of the expression of known target genes (*c-Fos*, *c-Jun* and *TGF-β1*) or control (*p21^CIP1^*). p38β_2_ and MKK6(Glu) were co-expressed in HeLa cells treated with MG-132. Relative gene expression was assessed in absence (control) or presence of ATF7-4 WT or mutant version. Data were normalized to hypoxanthine-guanine phosphoribosyltransferase (HPRT) gene expression. Results are the average of three independent experiments. Standard deviations and statistical significance by Student t-test are shown: NS = nonsignificant, **p<0.01, ***p<0.001.

To further confirm the implication of the proteasome in the phospho-ATF7-4 degradation, we co-expressed ATF7-4 and His-tagged ubiquitin in HeLa cells treated with MG-132. His-tagged proteins, i.e. ubiquitin and ubiquitin-conjugated proteins, were purified on nickel beads and analyzed by immunoblotting with specific antibodies. Rabbit antiserum recognizing phosphorylated ATF7-4 (pATF7-4) enabled us to detect a specific pattern of poly-ubiquitinated phospho-ATF7-4 proteins (Figure 5D, lane 2). These results demonstrate that phosphorylated ATF7-4 are substrates for poly-ubiquitination.

Taken together, these results strongly suggest that ATF7-4 is specifically degraded via the ubiquitin-proteasome 26S pathway when it is phosphorylated, whereas ATF7-FL is not.

### ATF7-4 downregulates the transcriptional activity of ATF7-FL and ATF2

We looked for a functional role of ATF7-4 isoform. Since ATF7-4 and ATF7-FL proteins share the same activation domain, but are located in two distinct cellular compartments, we tested whether ATF7-4 could have an effect on ATF7-FL transcriptional activity or act as a negative regulator form of ATF7-FL. To answer this question, we assessed the transcriptional activity of ATF7-FL under various conditions. In order to circumvent a potential interference of the endogenous ATF proteins, we used an assay based on a luciferase reporter controlled by Gal4 fusion proteins [35]. ATF7 and ATF2 N-terminal sequences were fused to the Gal4 DNA-binding domain (Figure 5A, a). The resulting fusion proteins were co-expressed in HeLa cells with increasing amounts of ATF7-4 wild-type (WT) or mutant versions (Figure 5A, b), and their transcriptional properties were monitored by measuring the luciferase activity in the cellular extracts. The ATF7-4 T51A T53A and ATF7-4 C9A T51A T53A derivatives are non-phosphorylable proteins, while the latter exhibits an additional mutation in the zinc finger motif that prevents the binding of different ATF7 partners, among which its TAF12 coactivator [8]. The activity of the Gal4-ATF7(2–82) moiety was clearly inhibited in the presence of increasing amounts of ATF7-4 WT and to a lesser extent by ATF7-4 T51A T53A, whereas it was unaffected by the ATF7-4 C9A T51A T53A protein (Figure 5B).

To determine whether this phenomenon is specific to ATF7 or has a wider impact, we then analyzed the effect of ATF7-4 on the transcriptional activity of ATF2 transcription factor, structurally and functionally related to ATF7. The impact of ATF7-4 on the transcriptional activities of Gal4-ATF2 and the unrelated Gal4-Elk1 factor as a control, was assessed by luciferase assays. Interestingly, ATF7-4 expression resulted in a strong inhibition of Gal4-ATF2(19–96) transcriptional activity (Figure 5C) but had no effect on Gal4-Elk1 activity (data not shown). Taken together, these results demonstrate that ATF7-4 acts as a negative regulator of the transcriptional activation mediated by members of the ATF7/ATF2 family.

We next investigated whether ATF7-4 was able to inhibit the expression of cellular genes whose promoters are regulated through CRE or TRE elements bound by ATF7 or ATF2 proteins (*c-Fos*, *c-Jun* and *TGF-β1* genes) [36], [37], or by Jun/Sp1 proteins (*p21^CIP1^* gene) [38]. To this end, ATF7-4 WT or mutant C9A T51A T53A version were co-expressed with activated p38β_2_ in HeLa cells treated with MG-132 to avoid any degradation of ATF7-4. The relative mRNA expression was assessed for these four genes by real-time quantitative RT-PCR with specific primers (Table S1). In contrast to ATF7-4 C9A T51A T53A (Figure 5D, black bars), ATF7-4 WT markedly and significantly reduced the p38β_2_-induced expression of *c-Fos*, *c-Jun*, and *TGF-β1* genes (Figure 5D, grey bars), whereas both had no effect on the *p21^CIP1^* gene expression level. These results further emphasize the ability of ATF7-4 to downregulate the expression of genes controlled by transcription factors of the ATF7/ATF2 family. The fact that ATF7-4 only exhibits its interfering effect when its N-terminal zinc finger motif (a protein-protein interaction domain) is intact strongly suggests that ATF7-4 could compete for a common partner that plays a major role in the transcriptional activation process.

### ATF7-4 specifically inhibits ATF7-FL and ATF2 phosphorylation

**Figure 6:**
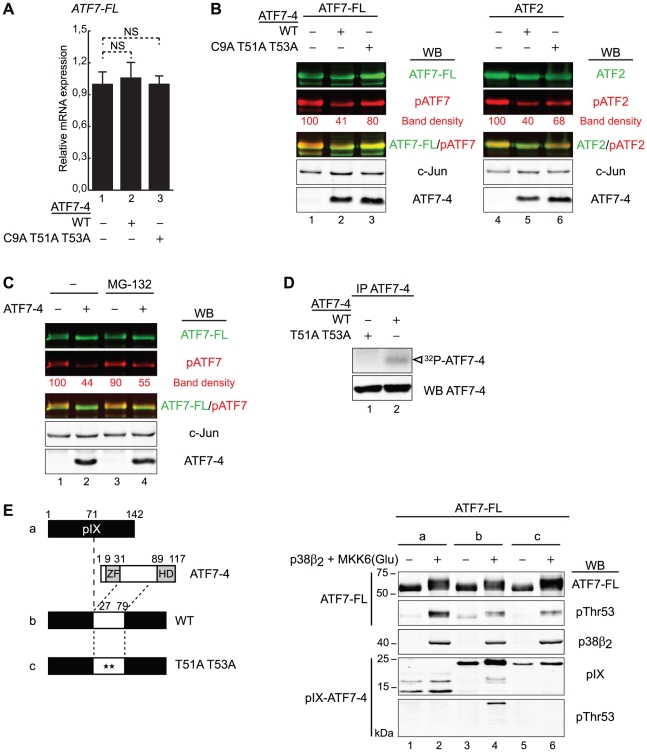
ATF7-4 impairs ATF7-FL/ATF2 phosphorylation by retaining a specific kinase. (A) Endogenous ATF7-FL relative mRNA expression levels were assessed by real-time RT-PCR in HeLa cells, in presence of overexpressed ATF7-4 WT or mutant derivative. Data were normalized to hypoxanthine-guanine phosphoribosyltransferase (HPRT) gene expression. Results are the average of two independent experiments. Standard deviations and statistical significance by Student t-test are shown: NS = nonsignificant. (B and C) The phosphorylation state of overexpressed ATF7-FL and ATF2 was established in presence of ATF7-4 WT or mutant version, in HeLa cells treated (C) or not (B) with MG-132 proteasome inhibitor. The analysis was performed on total cell extracts by western-blotting (WB) with specific antibodies as indicated. Images of total ATF7-FL/ATF2 (green channel) and phosphorylated forms (red channel) were merged with LI-COR Odyssey software. c-Jun levels were used as a loading control. The relative band density of phosphorylated forms was quantified and normalized to c-Jun band density. Results of representative experiments are shown. (D) HeLa cells overexpressing ATF7-4 or mutant version were treated with MG-132. ATF7-4 and the associated proteins were immunoprecipitated with anti-ATF7-4 specific antibody, and assayed for a kinase assay with radiolabelled [*γ*-^32^P]ATP. The analysis was performed by autoradiography (top panel) and WB (bottom) in parallel. The white arrow indicates radiolabelled ATF7-4. (E) The phosphorylation state of overexpressed ATF7-FL was analyzed in presence of pIX (a) or pIX-ATF7-4 fusion proteins with the docking site of ATF7-4 for kinases, either WT (b) or mutant version (c). HeLa cells were co-expressed with p38β_2_ and MKK6(Glu). Cell lysates were analyzed by WB to detect the specific phosphorylation of ATF7-FL (two upper panels) and pIX-ATF7-4 fusion proteins (two lower panels).

To decipher the mechanism leading to the inhibition of ATF7-FL and ATF2 transcriptional activities, we first tested whether ATF7-4 could have an effect on ATF7-FL expression *per se*. To this end, ATF7-FL relative mRNA expression in HeLa cells was measured by real-time quantitative RT-PCR in the presence of ATF7-4 WT or its C9A T51A T53A derivative. No significant differences in ATF7-FL expression level could be detected (Figure 6A), clearly indicating that ATF7-4 impairs the ATF7-FL/ATF2 activities by another mechanism than by inhibiting their own mRNA expression.

The decrease of transcriptional activity could result from either a downregulation at the protein level or a deficiency in the phosphorylation process. Thus, the overall level and phosphorylation state of ATF7-FL and ATF2 proteins were determined by western-blotting in HeLa cells co-expressing ATF7-FL or ATF2 with ATF7-4 WT or its mutant version. To clearly delineate the different species of ATF7-FL/ATF2 proteins, the overall protein signal and the Thr53/Thr71 phospho-specific signal were merged. In absence of any activation, c-Jun protein level was stable and used as a loading control. Upon ATF7-4 WT expression, we observed a 60% reduction of the phospho-specific signals and a concomitant loss of mobility-shifted forms (Figure 6B, lanes 2 and 5), while the overall amounts of ATF7-FL and ATF2 proteins were only slightly affected. By contrast, the phosphorylation levels of these two proteins was much less reduced by co-expression of the ATF7-4 C9A T51A T53A mutant (Figure 6B, lanes 3 and 6). Thus, ATF7-4 specifically impairs ATF7-FL/ATF2 phosphorylation on residues Thr53/Thr71 respectively.

To further elucidate the process involved, we tested whether these phosphorylated forms could be degraded, by repeating the experiment under a MG-132 treatment. The loss of ATF7-FL Thr53 phosphorylation was not significantly influenced by this treatment (Figure 6C), indicating that a proteasome-independent process is involved.

Furthermore, co-expression of ATF7-4 had no effect on the subcellular localization of either total or phosphorylated ATF7-FL species (Figure S3), ruling out any relocation induction.

We have previously demonstrated that phosphorylation and sumoylation of ATF7-FL are mutually exclusive [23]. It was therefore of interest to examine whether ATF7-4 induced a change in the sumoylation status of ATF7-FL. We showed that the decrease of ATF7-FL phosphorylation achieved by increasing amounts of ATF7-4 was not paralleled by a concomitant increase of ATF7-FL sumoylation (Figure S4).

Together, these results clearly demonstrate that ATF7-4 does not influence ATF7-FL stability or subcellular localization, whereas it specifically affects the phosphorylation of ATF7-FL/ATF2 on residues Thr53/Thr71 respectively, which is the first event leading to their activation.

### ATF7-4 binds to a Thr53-phosphorylating kinase in the cytoplasm

ATF7-FL can be phosphorylated by associated protein kinase activities under *in vitro* assay conditions [22], [39]. In a search of a kinase activity that could be associated with the ATF7-4 protein, we immunoprecipitated ATF7-4 from HeLa cells that had been transfected with vectors expressing either ATF7-4 WT protein or the T51A T53A derivative. The immunoprecipitates were then incubated under *in vitro* kinase assay conditions. *In vitro* phosphorylation of ATF7-4 WT was detected (Figure 6D, lane 2), while no labelling of the T51A T53A mutant was found (Figure 6D, lane 1), indicating that a protein kinase targeting Thr51 and/or Thr53 was immunoprecipitated with ATF7-4. Thus, a protein kinase is associated to ATF7-4 in the cytoplasm.

To address whether this phenomenon is responsible for ATF7-4 interference in ATF7-FL phosphorylation, we decided to relocate ATF7-4 and examine whether it was still phosphorylated and able to compete for ATF7-FL phosphorylation. We thus constructed pIX-ATF7-4 fusion proteins with the central region of ATF7-4 (residues 27–79 of either the WT or T51A T53A version) inserted within the sequence of adenovirus protein IX (pIX) (Figure 6E). pIX is a protein that contributes to the Ad5-induced reorganization of the host cell nuclear ultrastructure by inducing the formation of nuclear inclusions into which proteins can be relocated [40], [41]. pIX can also be used as a platform for the incorporation of heterologous proteins [42]. ATF7-FL was co-expressed with the pIX-ATF7-4 fusion proteins and activated p38β_2_ MAPK in HeLa cells, and their phosphorylation states were analyzed by western-blot. We could detect pIX-ATF7-4 phosphorylation on Thr53 (Figure 6E, lane 4), demonstrating that this central region of ATF7-4 functions as a docking site for an associated kinase. As expected, the expression of pIX-ATF7-4 WT resulted in a decrease of ATF7-FL phosphorylation on Thr53 (Figure 6E, lane 4), confirming that ATF7-4 competes with ATF7-FL for the binding to the kinase that specifically phosphorylates the Thr53 residue in the nucleus. Interestingly, the T51A T53A non-phosphorylable mutant had the same effect (Figure 6E, lane 6), indicating that it is also able to associate to this kinase, in contrast to the C9A T51A T53A derivative further mutated in the zinc finger interaction motif.

Altogether, these results suggest that ATF7-4 inhibits ATF7-FL/ATF2 phosphorylation and subsequent activation by retaining a Thr53/Thr71-specific kinase in the cytoplasm.

## Discussion

**Figure 7:**
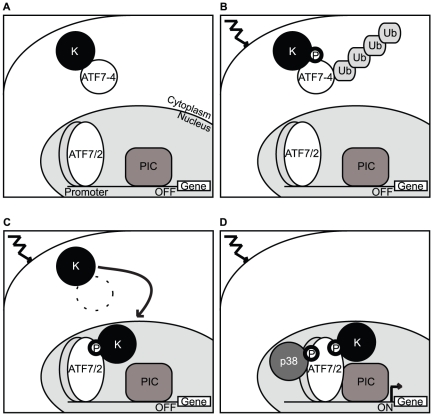
Model illustrating the regulatory role of ATF7-4. (A) Under resting conditions, ATF7-4 retains in the cytoplasm a kinase (K) that is able to phosphorylate ATF7-FL/ATF2 on Thr53/Thr71 respectively, preventing any transcription activity on their target genes. (B) The stimulus-induced phosphorylation of ATF7-4 promotes its poly-ubiquitination and degradation. (C) Our model proposes that the kinase is subsequently released, enters the nucleus and phosphorylates ATF7-FL/ATF2. (D) This first phosphorylation event is necessary for stress-activated p38 recruitment and ATF7-FL/ATF2 phosphorylation on Thr51/Thr69 respectively. The double phosphorylated forms associate efficiently with preinitiation complex (PIC) and are transcriptionally active.

This study extends our previous comprehension of the molecular mechanisms leading to the regulation of the transcriptional activation of the ATF7/ATF2 transcription factors family. We identified a short alternatively spliced variant of ATF7 that encodes a cytoplasmic protein, ATF7-4. This protein prevents ATF7-FL and ATF2 activation, very likely by retaining in the cytoplasm the kinase responsible for the first ATF7-FL/ATF2 phosphorylation event. When ATF7-4 gets phosphorylated, it is poly-ubiquitinated and degraded by a proteasome 26S-dependent pathway. We propose that the sequestered kinase is subsequently released, that it enters into the nucleus and phosphorylates the ATF7-FL/ATF2 proteins. As previously described, this priming phosphorylation enables the recruitment of p38 kinases for ATF7-FL/ATF2 activation, which promotes their association to pre-initiation complexes on promoters and transcription of their target genes (Figure 7) [23]. In spite of its relatively low level of expression due to its protein instability, ATF7-4 is highly conserved in mammalian species (except mouse and rat) (Figure 1C) and expressed in a wide range of tissues (Figure 2). Considering its sensitivity to proteolytic degradation (Figure 4C and D) and its subcellular localization (Figure 3), we are confident that ATF7-4 plays an important role in the control of transcription.

ATF7-4 is a specific variant resulting from the ATF7 gene expression that has no equivalent in the ATF2 gene. Although human ATF7 and ATF2 genes map on different chromosomes (ATF7 12q13; ATF2 2q32), the analysis of their genomic organization in terms of intron/exon distribution indicates that both genes have a similar structure and may have arisen by duplication of a common precursor. However, their intronic sequences between exons 4 and 5 are completely different. The ATF7 exon 4, in contrast to its ATF2 counterpart, is adjacent to an additional alternative exon (exon 4a) containing an open-reading frame that is followed by a polyadenylation sequence (AATAAA), giving rise to a functional messenger encoding ATF7-4 protein. Nevertheless, the molecular mechanism leading to exon 4a inclusion and ATF7-4 generation remains unclear. Based on various examples of pre-mRNA 3′ end processing regulation, different mechanisms of action have been proposed, including a kinetic competition between the poly(A) signal recognition, splicing and transcription, directly correlated with the rate of their respective machinery assembly [43], [44], [45]. As a result, ATF7-4 encompasses the N-terminal moiety of the ATF7 activation domain fused in frame to a specific C-terminal hydrophobic domain (Figure 1B and C), the latter mediating its cytoplasmic localization (Figure 3). Furthermore, bioinformatic analyses indicate that this domain shares all the characteristics of a transmembrane helix [46], the N-terminal part of the protein being in the cytoplasm. These data are consistent with the fact that we isolated the TRAPalpha/SSR1 protein as an ATF7 activation domain-interacting partner in a yeast two-hybrid screening (B. Chatton, unpublished). The SSR1 protein is a single-spanning membrane protein of the endoplasmic reticulum (ER) that is found in proximity to nascent polypeptide chains translocating across the membrane [47]. Although the ability of ATF7-4 to anchor in cytosolic membranes with the help of SSR1 has not yet been demonstrated, it is tempting to speculate that this process might contribute to the control of ATF7-4 subcellular localization under specific conditions.

Tissue-specific differential splicing or promoter usage also give rise to ATF2 variants, but none of them exhibits a dominant negative activity. Most of these variants are ubiquitously expressed, although some are particularly enriched in specific tissues. Three ATF2 isoforms are expressed in murine T-cells: CRE-BP1, CRE-BP2 and CRE-BP3. Although these isoforms differ by N-terminal or internal residues, they all retain the bZIP domain, suggesting that their transcriptional activity is conserved, even if their regulation is intrinsically different [48], [49]. Another ATF2 splicing variant is expressed in myometrial tissues during pregnancy/labor (GenBank: AY029364.1) [50]. ATF2-small (ATF2-sm) only comprises the first two and last two exons, lacking therefore the most part of the functional domains, i.e. the sequence encompassing the two threonine residues 69 and 71 of the activation domain and the bZIP domain. Nevertheless, it was intriguingly described to exhibit a CRE-binding activity and to be transcriptionally active to an equivalent degree to that of full length ATF2 [50]. Yet, no inhibitory effect has been associated to this variant. Therefore, among all these described isoforms, ATF7-4 is the only negative regulator form of the ATF7-FL/ATF2 transcription factors family characterized to date, further highlighting its considerable importance.

The ATF7-4 phosphorylation process is consistent with the previously described ATF7-FL dual phosphorylation (Figure 4B) [23]. Indeed, this two-step mechanism also involves a priming, yet unidentified, kinase activity that phosphorylates residue Thr53. It is followed by the secondary phosphorylation of Thr51 by activated p38β_2_ kinase. Our data suggest that ATF7-4 could sequester in the cytoplasm the unknown priming kinase thereby preventing ATF7-FL and ATF2 phosphorylation of Thr53 and Thr71 (Figure 6). It has been well documented that ATF2 is activated through phosphorylation on threonine residues 69/71 by stress-activated protein kinases JNK or p38 [17], [18], [51], [52], [53]. ATF2 is also activated by growth factors via the Ras-MEK-ERK pathway, for initial Thr71 phosphorylation, in concert with the RalGDS-Src-p38 kinases, for subsequent Thr69 phosphorylation, thus involving cooperation between distinct signaling pathways for its transcriptional activation [19], [20], [21]. However, based on the effects of specific inhibitors (U0126 or SB203580) and the results of transfection experiments in Jnk1 and Jnk2 deficient fibroblasts, we ruled out that ERK or JNK MAP kinases could be involved in the first ATF7-FL phosphorylation event on residue Thr53 [23]. It appears therefore that a distinct priming kinase activity is responsible for this phosphorylation event in both ATF7 and ATF2.

The present study offers clear evidence that ATF7-4 functions as a negative regulator counterpart of ATF7-FL and ATF2 activities. Such entities have provided substantial insights into the molecular mechanisms of action of a number of protein families, including hormone or growth factor receptors and oncogenes. In agreement with our findings, Z. Ronai and collaborators have demonstrated the ability of a 51-residue polypeptide derived from the ATF2 transactivating domain (ATF2^50–100^) to alter melanoma growth and metastasis capacity *in vivo* [12], [54], [55]. Given the strong homology between ATF2 and ATF7, ATF7-4 might thus be considered as the cellular counterpart of this synthetic peptide. ATF7-4 and ATF2^50–100^ peptide share functional characteristics, including their cytoplasmic localization, as well as the ability to downregulate ATF2 transcriptional activity and to associate with kinases [12], [55]. In addition, the ATF2^50–100^ peptide has been shown to induce the relocation of ATF2 and c-Jun proteins to the cytoplasm and a concomitant increase of c-Jun expression, without significantly altering the phosphorylation of ATF2 [54]. However, in presence of ATF7-4, we observed neither a relocation of ATF7-FL and c-Jun nor an accumulation of the latter protein in HeLa cells under our experimental conditions (Figure S2). Thus, ATF7-4 and ATF2^50–100^ peptide both control ATF2 activity, but distinct mechanisms of action may be involved. This discrepancy may be related to the fact that ATF7-4 contains two additional functional domains, i.e. the zinc-finger motif and the C-terminal hydrophobic domain, which are implicated in protein-protein interactions and the control of ATF7-4 subcellular localization respectively.

The mechanism that we uncovered enables us to delineate the docking site of ATF7 for kinases. We found that the ATF7-4 (27–79) domain is able to interact with the priming kinase whether the WT or T51A T53A versions were used (Figure 6E). These results indicate firstly that the priming kinase docking site is located within the 27–79 region of ATF7-4, a domain matching precisely the ATF2^50–100^ peptide, and secondly that the two threonine residues are not essential for the binding. Further corroborating our study, other proteins have been described to interact with MAPK in the cytoplasm and prevent their translocation into the nucleus. For instance, PEA15 and protein tyrosine phosphatase PTP-SL, which retain MAPK in the cytoplasm [56], [57], or the regulatory protein TAB1 that competes with MKK3 for p38 interaction, binds to p38 and prevents its nuclear localization [58].

The phosphorylation of ATF7-4 induces its degradation (Figure 4). The general principles of phosphorylation-dependent ubiquitination are clearly understood. Phosphorylation on specific residues can result in the generation of a motif, known as phosphodegron, which is specifically recognized by F box proteins within E3 ligase complexes (for a review see [1]). However, the mechanism governing phosphorylation-dependent degradation of ATF7-4 remains unclear. Its phosphorylation by the priming kinase may either activate a specific cytosolic E3 ligase responsible for ubiquitin conjugation or promote its recognition by the E3 ligase while creating a phosphodegron. The involvement of a cytoplasm-specific E3 ligase could explain why under these conditions phosphorylated ATF7-4 is degraded whereas nuclear ATF7-FL and ATF2 are not. Moreover, previous studies have provided evidence that under resting conditions, nuclear ATF2 is unstable and degraded. Upon stress induction, this protein is phosphorylated and activated, leading to its stabilization and protection from degradation until its subsequent downregulation by phosphatases [59], [60]. This model is consistent with our findings, suggesting that phosphorylation induces a switch from ATF7-4 primacy to ATF7-FL/ATF2 action.

In summary, our results demonstrate in our cellular model (HeLa cells) a novel important role for a cytoplasmic isoform of a transcription factor in inhibiting the activity not only of its nuclear counterpart (ATF7-FL), but also of a distinct one (ATF2). We provide novel mechanistic insights into the temporal and spatial regulation of these transcription factors, through the control of their specific kinase location. The kinases remain however to be identified. It will also be of interest to examine whether the cytoplasmic interaction of this kinase with ATF7-4 also interferes with other cellular processes, which would further extend the functional importance of ATF7-4.

## Materials and Methods

### Expression vectors

The recombinant human ATF7 isoforms used in this study are ATF7-4 and ATF7-1, the latter referred to as ATF7-FL, keeping the amino acid coordinates of the largest protein (ATF7-3, 494 residues) [8]. The full sequences of the ATF7-4 transcript (787 nucleotides) and protein (117 amino acids) have been given the GenBank and Swissprot accession numbers BC042363 and Q8IVR8 respectively. The pG4-ATF7(2–82) and pG4-ATF2(19–96) plasmids encode the DNA-binding domain of the yeast Gal4 protein fused to the human activation domain of either ATF7-FL (amino acids 2–82) or ATF2 (amino acids 19–96), as previously described [39], [52]. The (17m5)-TK-Luc reporter [61] contains the luciferase gene driven by the thymidine kinase promoter and five Gal4 binding sites. The cDNA of ATF7-FL, ATF7-4, ATF2 and mutant versions were also inserted into the pXJ plasmid, under the control of the cytomegalovirus (CMV) promoter [62], generating the px-ATF series. The pXJ-pIX-ATF7-4 vector and derivative encode the pIX adenoviral (Ad 5) protein fused to a central moiety of ATF7-4 (amino acids 27–79). The peGFP-ATF7-4 vector and mutant version encode the eGFP protein fused to the C-terminal part of ATF7-4 (amino acids 89–117). Plasmids encoding dominant active MKK6 [referred to as MKK6(Glu)] [18], p38β_2_ [63], and 6His-Ubiquitin [64] were kindly provided by J. Raingeaud, J. Han and M. Treier respectively. Point mutations and deletions were generated by oligonucleotide-directed mutagenesis using *Pfu* DNA polymerase (Fermentas), PCR amplification and restriction endonuclease digestion of the appropriate DNA fragments. All constructs were verified by DNA sequencing.

### Cell culture, RNA interference, transfection, extract preparation and fractionation

HeLa, H1299, U-2 OS and MEF cells were grown as monolayers in Dulbecco's modified Eagle's medium (DMEM) supplemented with 4.5 g/L glucose and 10% fetal calf serum (FCS) [5] [65] [39] [66]. Raji [67], 501Mel [68] and HTR-8/SVneo [69]cells were maintained in RPMI 1640 medium containing 10% FCS. HEK[70], HCT 116 [71], and HeLa-SUMO cells were grown in DMEM supplemented with 1 g/L glucose and FCS, 10%, 7.5% and 5% respectively. A549 [72] and 1205Lu cells were cultured respectively in DMEM/F12 medium supplemented with 10% FCS, and in complete tumor medium W489, a 3∶1 mixture of MCDB153 and Leibovitz L15 supplemented with 4% FCS. HeLa-SUMO cells, constitutively expressing high levels of 6His∶Myc∶epitope-tagged human SUMO-1 protein, were kindly provided by P. O'Hare [73]. HTR-8/SVneo, 1205Lu [74] and 501Mel cells were kindly provided by V. Sapin, G. Mengus and I. Davidson respectively.

Cells were transiently transfected using TransIT®-LT1 reagent (Mirus Bio Corporation, Euromedex) according to the manufacturer's protocol. After 28 h, cells were harvested in phosphate-buffered saline (PBS) and resuspended in RIPA buffer [150 mM NaCl, 20 mM Tris-HCl pH 7.8, 0.1% SDS, 1% Triton X-100, 0.5% sodium deoxycholate, 1 mM dithiothreitol [DTT], 1 mM sodium orthovanadate, Protease Inhibitor Cocktail (Sigma)]. After 30 min on ice, the resulting crude suspension was cleared by centrifugation for 15 min at 10,000 g. Cells at 30–50% confluence were transfected with 15 nM of control or specific siRNA (siATF7-4, 5′-AA-CATGGGCTTAGTAGAGTAA-AA-3′, Ambion, Austin, TX) using HiPerFect reagent (Qiagen) and harvested after 48 h.

In Figures 6B and C, cellular proteins were extracted by boiling cell pellets 30 min in SDS sample buffer [125 mM Tris-HCl pH 6.8, 40% glycerol, 8% SDS, 200 mM DTT, 0.02% bromophenol blue] and the lysates were then homogenized by a 4 min-sonication in a cold water bath (Bioruptor® Next Gen, Diagenode).

For the phosphatase treatment, cell lysates were incubated for 1 h at 37°C with 10 units of calf intestinal phosphatase (CIP, New England Biolabs) in 50 µL of reaction buffer [50 mM Tris-HCl pH 7.8, 50 mM KCl, 10% glycerol and 1 mM DTT] supplemented with 50 mM EDTA for CIP inhibition where indicated. Cells treated with proteasome inhibitor were incubated for 2 h with 30 µM MG-132 (Calbiochem).

For biochemical fractionation, cellular proteins were extracted in lysis buffer [10 mM Hepes pH 7.9, 1.5 mM MgCl_2_, 10% glycerol, 0.1% Nonidet P-40, 0.1 mM EGTA, 0.5 mM DTT, 1 mM N-ethyl-maleimide (NEM), Protease Inhibitor Cocktail] as described [75]. Lysates were centrifuged and the supernatant was retained (cytoplasmic fraction). The remaining pellets were incubated for 1 h on ice in lysis buffer supplemented with 300 mM NaCl. The resulting lysates were cleared by centrifugation (nuclear/euchromatin fraction). The pellets were digested for 30 min at 30°C with 300 units DNase (Sigma) in digestion buffer [20 mM Tris-HCl pH 7.4, 5 mM MgCl_2_, 250 mM sucrose, 1 mM NEM, Protease Inhibitor Cocktail]. Extracts were centrifuged and the supernatants were retained (heterochromatin fraction).

### Luciferase assay

HeLa cells (2.10^5^ cells per well in six-well plates) were transfected using ExGen 500 reagent (Euromedex) with a mixture containing 0.5 µg of the luciferase reporter, 0.2 µg of the pG4-ATF expression vector, and up to 0.3 µg of the px-ATF7-4 plasmid. The total amount of DNA was adjusted to 1 µg with empty vector. After 24 h, cells were washed in PBS and incubated for 30 min in lysis buffer [1% Triton X-100, 25 mM glycyl-glycine pH 7.8, 15 mM MgSO_4_, 4 mM EGTA]. The resulting cell lysate was assayed for luciferase activity (normalized by protein concentration) using a Berthold Centro LB 960 luminometer as previously described [76], [77]. At least two independent transfections were carried out in triplicate and the results always agreed within 10%. Error bars show standard deviations (n = 3).

### Antibodies

A rabbit antiserum directed against ATF7FL or ATF7-4 (epitope 42–60) with high affinity for phosphorylated threonine 51 and threonine 53 (pATF7), and monoclonal antibodies specifically recognizing ATF7-FL isoforms (ATF7-FL, 2F10) or phosphorylated ATF7 on residues threonine 51 (pThr51, 2H1), threonine 53 (pThr53, 1H9) and threonine 112 (pThr112, 2F3) have been previously described [23], [39]. To generate specific anti-ATF7-4 monoclonal antibodies, a peptide [KKLVVFRPRLFLL] corresponding to amino acids 96–108 was synthesized, coupled to ovalbumin as a carrier protein and used for mouse immunization. Immunization and monoclonal antibody production were essentially as reported by [78]. The specificity of the selected antibodies was verified by comparing their immunoreactivity against ATF7-4 versus ATF7-FL as previously described [23], [79]. These monoclonal antibodies directed against the C-terminal part of ATF7-4 specifically recognize non-phosphorylated ATF7-4 (Figure S1). Mouse anti-GFP (2A3), mouse anti-Gal4 (3GV2) and rabbit anti-pIX [80] antibodies have been previously described. Rabbit anti-c-Jun (H-79), mouse anti-ATF2 (F2BR1) and goat anti-p38ß_2_ (C-16) antibodies were purchased from Santa Cruz Biotechnology. Rabbit antiserum specifically recognizing phosphorylated ATF2 (pATF2) and rabbit anti-ubiquitin were obtained from Abcam (ab28848) and Sigma (U5379) respectively. Anti-SUMO-1, -Paxillin, -Lamin and -HP1 antibodies were kindly provided by J.S. Seeler and A. Hamiche.

### Ni-NTA agarose affinity purification, immunoprecipitation and western-blotting

Ni-NTA agarose affinity purification and immunoprecipitations were carried out as previously described [23] [8]. For western-blotting, proteins were electrotransferred onto nitrocellulose and reacted with specific primary antibodies (see above). Bound antibodies were detected with IRDye800-conjugated anti-mouse (green channel) and IRDye700-conjugated anti-rabbit (red channel) secondary antibodies (ScienceTec). Membranes were scanned with the LI-COR Odyssey infrared imaging system. Anti-mouse and anti-rabbit signals were merged with the Odyssey v3.0 software (LI-COR Biotechnology, ScienceTec). In all cases, at least three independent transfections were carried out and the results always agreed within 10%. The results of typical experiments are shown in the figures.

### Quantitative RT-PCR

The tissue-specific expression pattern of ATF7-4 was investigated by quantitative PCR on a RapidScan™ gene expression cDNA panel (OriGene Technologies) containing cDNA from 24 different human tissues normalized to β-actin gene expression. In the other experiments, total RNA was extracted using TRIzol® reagent (Invitrogen). Reverse-transcription was performed with 1 µg of total RNA using AMV reverse-transcriptase (Fermentas) and random hexamer primers as specified by the manufacturer. Total RNA of WRO and BCPAP thyroid cell lines were kindly provided by C. Ferraro-Peyret. Quantitative real-time PCR was performed with 10 ng of cDNA template on the StepOne Plus™ system (Applied Biosystems, Life Technologies) according to the manufacturer's protocol, using Power SYBR® Green and TaqMan® Universal PCR Master Mixes (Applied Biosystems). The sequences of the TaqMan® probes designed for specific ATF7-FL and ATF7-4 amplification, and the primer pairs used are shown in Table S1. Data were normalized to β-actin or hypoxanthine-guanine phosphoribosyltransferase (HPRT) gene expression. Results shown are representative of at least two independent experiments performed at least in triplicate. Error bars show standard deviations (*n* = 3). Statistical significance was determined by using Student t-tests.

### Immunofluorescence staining and confocal imaging

Immunofluorescence experiments were carried out as previously described [81]. Briefly, HeLa cells were grown on a Lab-Tek® Chamber Slide (Nunc) and fixed with 4% paraformaldehyde, 28 h after transfection. Cells were then permeabilized with 0.5% Triton X-100 in PBS and aspecific sites were saturated with 20% fetal calf serum in PBST [0.1% Triton X-100 in PBS]. Cells were incubated with specific primary antibodies diluted in PBST and directed against ATF7-FL (2F10), ATF7-4 (rabbit antiserum) and GFP (2A3). Three PBS washes were performed before incubating the cells with Alexa 488- or Cy3-conjugated anti-rabbit or mouse secondary antibodies (Molecular Probes, Invitrogen) diluted in PBST. Nuclei were counterstained with Hoechst 33258 dye (Sigma) and the preparations were mounted in ProLong® Gold medium (Invitrogen). Cells were analyzed by confocal laser-scanning microscopy (Leica). Quantification of nuclear and total fluorescence intensities was performed using ImageJ on at least 22 images per condition. Error bars show standard deviations (*n* = 22). Statistical significance was determined by using Student t-tests.

### Kinase assay

ATF7-4 and the associated proteins were immunoprecipitated with the monoclonal anti-ATF7-4 antibody. The proteins adsorbed to the protein G-sepharose beads were incubated for 40 min at 30°C in 40 µL of kinase buffer containing 20 µCi of [*γ*-^32^P]ATP (3000 Ci/mmol), 50 mM Tris-HCl pH 7.5, 10 mM MgCl_2_, 0.1 mM EGTA and 1 mM DTT. Cold ATP (1 mM) was added for the last 10 min to catalyze the reaction. The beads were then washed twice with the same buffer but lacking the radiolabelled nucleotide. Proteins were then dissociated by boiling for 5 min in 25 µL of SDS sample buffer, resolved by SDS-polyacrylamide gel electrophoresis (PAGE) [82] and electrotransferred to a nitrocellulose membrane. The ^32^P-labelled proteins were revealed by autoradiography and total proteins were analyzed by western-blotting as described [39].

## Supporting Information

Figure S1 **Characterization of the specificity of anti-ATF7-4 antibodies.** ATF7-4 was overexpressed in HeLa cells as indicated. Cell lysates were immunoprecipitated (IP) with different antibodies recognizing ATF7-4. Affinity-purified proteins were then analyzed by western-blot (WB) using anti-ATF7-4 or anti-pThr53 antibodies. Rabbit antiserum and anti-pThr51, anti-pThr53 monoclonal antibodies (lanes 2, 5 and 6 respectively) specifically recognize phosphorylated forms of ATF7-4, whereas the anti-ATF7-4 monoclonal antibody targets the non-phosphorylated form (lane 3). The anti-ATF7 Nt antibody directed against the N-terminal moiety of ATF7 was used as a control to facilitate the characterization.
(EPS)

Figure S2 **Pattern of expression of ATF7-FL transcripts.** Real-time RT-PCR analysis of ATF7-FL gene expression profile (A) in a 24 human tissue-cDNA array and (B) in a selection of human transformed cell lines. (A) PBL stands for peripheral blood leucocytes. (B) Data are the average of at least five independent experiments (standard deviations are shown). Data were normalized to β-actin gene expression.
(EPS)

Figure S3 **ATF7-FL subcellular localization is not affected by ATF7-4.** ATF7-FL was overexpressed in HeLa cells in presence of ATF7-4 as indicated. Lysates were fractionated into cytoplasm, nucleus/euchromatin and heterochromatin fractions, which were further analyzed by western-blot (WB). Images of total ATF7-FL (green channel) and phosphorylated forms (red channel) were merged with LI-COR Odyssey software. c-Jun, Lamin A/C, Paxillin and HP1α/β were used as loading and fractionation controls.
(EPS)

Figure S4 **ATF7-FL sumoylation is not impaired by ATF7-4.** (A) Schematic representation indicating the location of the consensus sumoylation site of ATF7-FL and the SUMO modification-defective mutant used (asterisk). (B) ATF7-FL or mutant version and an increasing amount of ATF7-4 were co-expressed in HeLa-SUMO cells. Cell lysates were immunoprecipitated with anti-ATF7-FL specific antibody and analyzed by western-blot (WB). The three upper panels are images of sumoylated ATF7-FL (green channel), total sumoylated proteins (red channel) and the overlay. In the three lower panels, images of total ATF7-FL (green channel) and phosphorylated forms (red channel) were merged. The overlays were performed with LI-COR Odyssey software.
(EPS)

Table S1 Primers and TaqMan probes used for real-time RT-PCR.
(EPS)
